# *Dendrobium officinale* polysaccharide decreases podocyte injury in diabetic nephropathy by regulating IRS-1/AKT signal and promoting mitophagy

**DOI:** 10.18632/aging.205075

**Published:** 2023-10-08

**Authors:** Huahua Li, Jin Zheng, Yacen Wu, Hong Zhou, Suli Zeng, Quanqing Li

**Affiliations:** 1 Department of Geriatric, Hunan Provincial People’s Hospital, The First Affiliated Hospital of Hunan Normal University, Furong, Changsha 410005, P.R. China; 2 Department of Rehabilitation, Hunan Provincial People’s Hospital, The First Affiliated Hospital of Hunan Normal University, Furong, Changsha 410005, P.R. China

**Keywords:** diabetic nephropathy, mitophagy, podocyte, oxidative stress, *dendrobium officinale* polysaccharide

## Abstract

Backgrounds: High glucose (HG) caused oxidative stress and mitochondrial dysfunction, resulting in insulin resistance in podocytes, a key mechanism of diabetic nephropathy. *Dendrobium officinale* polysaccharide (DOP) was able to improve insulin resistance and antioxidant capability.

Objective: The purpose of this study is to explore the mechanism by which DOP decreases the podocyte injury induced by HG.

Methods: MPC5 cells were treated with HG, DOP, and IRS-1/2 inhibitor NT157. Afterwards, glucose consumption, generations of ROS and MDA were measured using the detection kits. Mitophagy was monitored using both MtphagTracyker and LysoTracker. The mitochondrial membrane potential was evaluated by JC-1 staining. DOP was also used in a mouse model of diabetes, with the measurements of urine albumin, blood creatinine and blood urea nitrogen.

Results: Treatment with DOP suppressed the HG-induced reduction of glucose consumption, the phosphorylation of IRS-1 (phospho Y632), AKT (phospho Ser473 and Thr308) and Nephrin. In addition, HG-induced augment of ROS and MDA, formation of γ-H2A.X foci and translocation of AKT to nucleus were inhibited by DOP. DOP enhanced mitophagy, which was associated with decreased mitochondrial membrane potential and ROS production. DOP conferred protective effect on podocyte in the diabetic mouse by reducing the albumin/creatinine ratio and blood urea nitrogen, and restoring Nephrin expression in podocytes.

Conclusions: DOP alleviates HG-induced podocyte injuryby regulating IRS-1/AKT signal and promoting mitophagy.

## INTRODUCTION

Diabetic nephropathy (DN) is a diabetes-related complication caused by prolonged hyperglycemia and has been the leading cause of end-stage renal disease in the world. The main characterizations of DN includes mesangial cell hypertrophy, persistent proteinuria, glomerular basement membrane thickening, and glomerular extracellular matrix accumulation [1, 2]. Among them, proteinuria, which results from the impairment of glomerular filtration barrier (GFB), is the earliest clinical manifestation in DN progression. Importantly, podocytes play a crucial part in the maintenance of glomerular filtration function, and pathogenesis of proteinuria [3, 4]. Besides, podocytes were validated to be the only insulin-sensitive cells in the GFB [5], with functional sites for insulin and complete signaling pathways [6, 7]. A previous report revealed that inability or reduction of insulin could stimulate glucose consumption in insulin target tissues [8]. Specially, the insulin responsiveness could be adjusted by podocytes through modulating the production of Nephrin, a crucial podocytes protein [9, 10]. Abnormal insulin signaling in podocytes have important impacts on the structure and function of kidney in diabetes [11–13]. Therefore, an in-depth investigation of insulin resistance (IR) in podocytes will make great significance for understanding of the pathogenesis of DN.

Previous investigations verified that there was a close association between IR and oxidative stress [14, 15]. Once the production of reactive oxygen species (ROS) is abnormally increased in the body, and cannot be effectively removed by the antioxidant system, it will result in an increase in oxidative stress level, and eventually lead to formation of IR. Besides, oxidative stress was reported to play important roles in inducing podocyte dysfunction, and promoting proteinuria occurrence [16]. A previous research indicated that oxidative stress with increased oxidation of free fatty acids (FFAs) and augmented production of superoxide induces the disorder of insulin signaling and changes of the insulin sensitivity [17]. Insulin can bind to the insulin receptor and phosphorylate IRS-1/2, which will lead to AKT phosphorylation, and further increase glucose consumption and ROS production [18].

Mitophagy dysfunction is connected to cellular oxidative stress and IR [19]. Mitophagy is essential for the maintenance of normal cell function and intracellular homeostasis. For instance, mitophagy inhibited high glucose (HG)-induced ROS generation [20] and podocyte apoptosis [21]. However, HG inhibited oxidative respiration in podocyte mitochondria and mitophagy [22], resulting in disruption of cellular energy supply, oxidative stress, and inflammation [23]. It has been reported that some herbal extracts, such as Icariin, Astragaloside II, and Orientin can influence podocyte mitophagy [21–23].

*Dendrobium officinale* (DO), as a kind of traditional Chinese medicine, was famous and prized for its well tonic efficacy. During the last few decades, numbers of investigations have found various pharmacological actions of *dendrobium officinale* polysaccharide (DOP), including anti-cancer, anti-inflammation, antioxidation, immunostimulatory and hypoglycemic functions [24, 25]. A study conducted by Zhao et al. manifested that DOP restrains IR in rats through suppressing Toll-like receptors and inflammatory response [26, 27]. Nevertheless, whether DOP achieved its efficacy in DN through modulating the oxidative stress and IR and mitophagy process of podocyte remains unknown.

This study aimed to explore the effects of DOP on oxidative stress, IR and mitophagy in the podocytes under the stimulation of HG *in vitro* and *in vivo.* The results provided theoretical basis for future clinical intervention of DN by DOP.

## RESULTS

### HG treatment induced oxidative stress and IR in MPC5 cells

**Figure 1:**
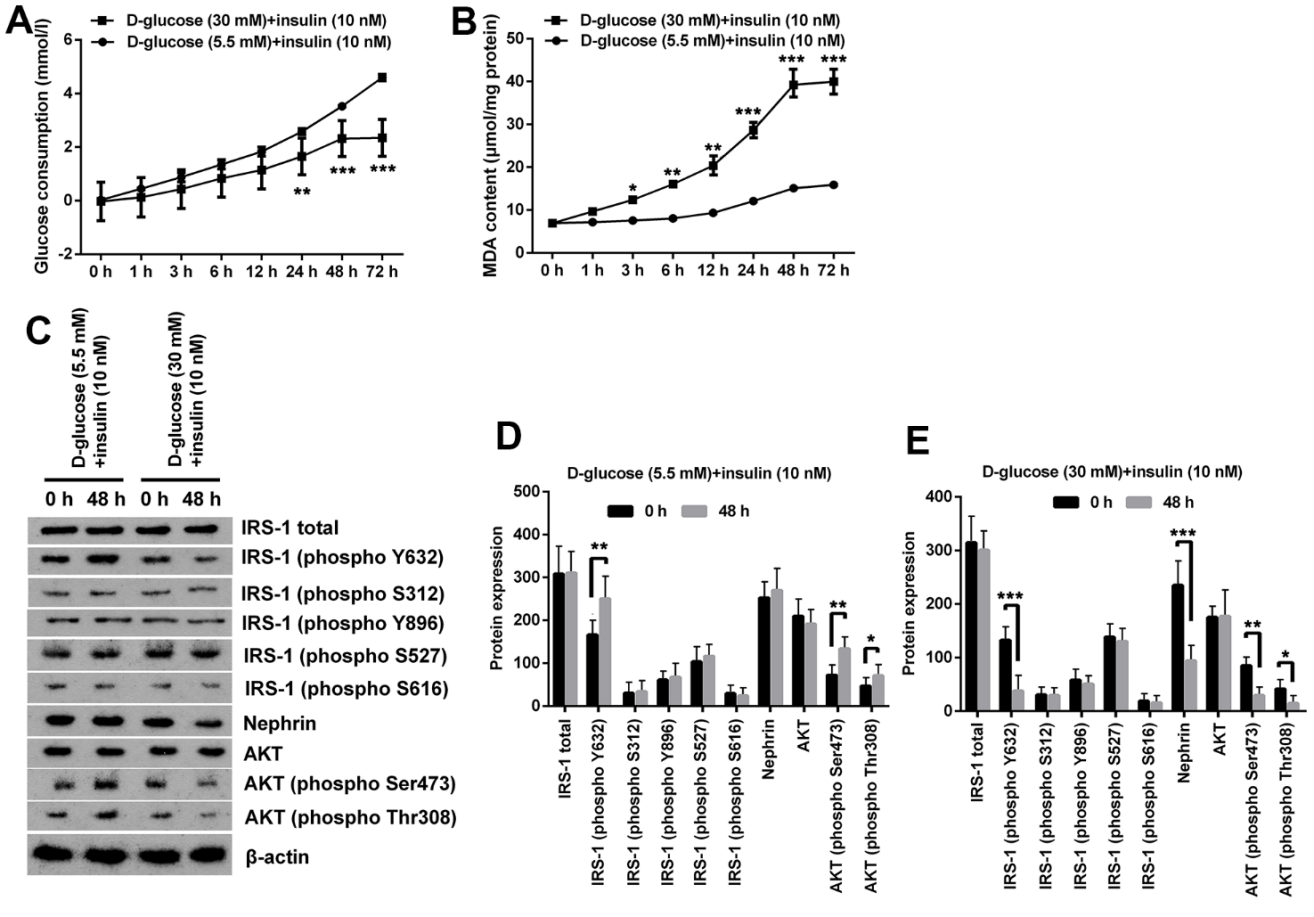
**High glucose treatment induced oxidative stress and IR in MPC5 cells.** (**A**) HG inducement distinctly reduced glucose consumption of MPC5 cells compared with normal glucose-treated group. (**B**) HG inducement notably augmented generations of malondialdehyde (MDA). (**C**–**E**) The phosphorylation of insulin receptor substrate (IRS)-1 (phospho Y632), AKT (phospho Ser473) and AKT (phospho Thr308) was markedly reduced by HG inducement. Besides, the expression of Nephrin was also notably decreased by HG inducement. (*P < 0.05; ** *p* < 0.01; *** *p* < 0.001, n=3).

In order to simulate the conditions of glomerular podocytes during DN, MPC5 cells were respectively exposed to normal/high D-glucose (5.5mM/30mM) for series of times, and then were treated with insulin. Afterwards, series of experiments related to oxidative stress and IR were performed. Results displayed in Figure 1A demonstrated that the glucose consumption was gradually increased along with time extension under normal D-glucose concentration treatment with HG notably decreased glucose consumption of MPC5 cells (*p* < 0.01 at 24h and *p* < 0.001 at 48 and 72h). Besides, glucose consumption of cells at 48 h and 72 h in HG group was basically the same. Measurement of MDA contents demonstrated that high glucose treatment notably augmented MDA production, which meant that oxidative stress was remarkably increased by HG treatment (*p* < 0.01, Figure 1B). Considering that there was no significant difference in MDA production between cells at 48 h and 72 h in HG group, therefore 48 h was selected as the timepoint for cell treatment in following experiments.

Additionally, expression of phosphorylated IRS-1, AKT and podocyte marker Nephrin revealed that normal glucose and insulin inducement notably augmented phosphorylation of IRS-1 (phospho Y632), AKT (phospho Ser473) and AKT (phospho Thr308) (*p* < 0.05 or *p* < 0.01), while there was no significant change observed on the expression other proteins including total IRS-1 and AKT (Figure 1C, 1D). Besides, HG and insulin inducement remarkably reduced the phosphorylation of IRS-1 (phospho Y632), AKT (phospho Ser473), AKT (phospho Thr308) and Nephrin (*p* < 0.05, *p* < 0.01 or *p* < 0.001), while had no significant influence on the expression of other proteins (Figure 1C, 1E). These results suggest that HG can induce IR and oxidative stress in MPC5 cells.

### DOP treatment distinctly relived high glucose-induced oxidative stress and IR of MPC5 cells

**Figure 2:**
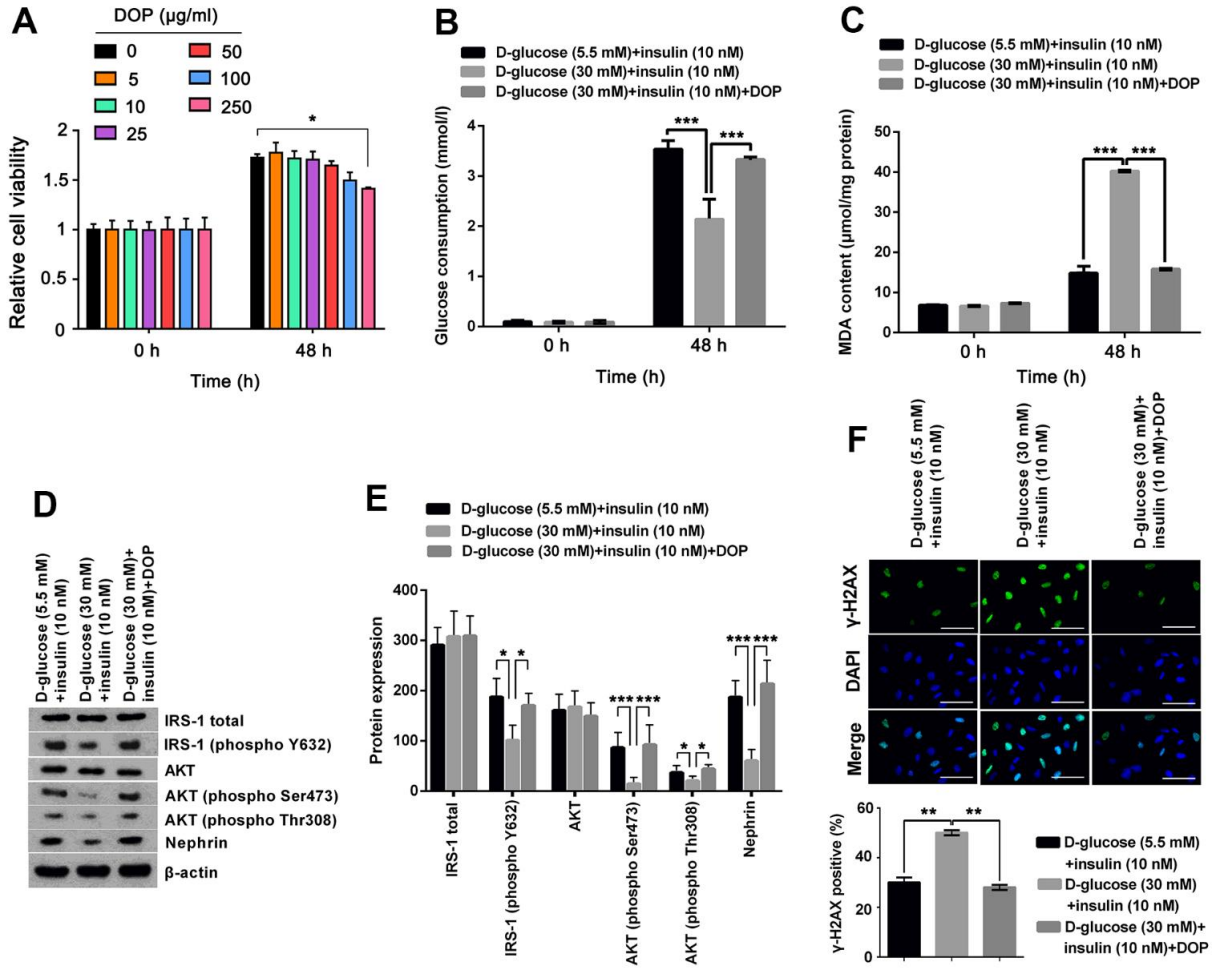
**DOP treatment distinctly relived HG-induced oxidative stress and IR of MPC5 cells.** (**A**) To determine the safety dosage of DOP, MPC5 cells were treated with various dosages of DOP (0, 5, 10, 25, 50, 100 and 250 μg/ml) for 48 hours. Cell viability was assessed by CCK-8 method. (**B**) DOP treatment notably augmented HG-reduced glucose consumption of MPC5 cells. (**C**) DOP treatment remarkably reduced HG-increased generations of malondialdehyde (MDA). (**D**, **E**) DOP treatment markedly relived HG-induced suppressive effects on the phosphorylation of insulin receptor substrate (IRS)-1 (phospho Y632), AKT (phospho Ser473), AKT (phospho Thr308) and Nephrin. (**F**) HG inducement markedly augmented the formation of γ-H2A.X foci, while DOP treatment notably reversed this effect. The bar in the figure indicates 10 μm (*p < 0.05; ** *p* < 0.01; *** *p* < 0.001, n=3).

To determine the safety dosage of DOP, MPC5 cells were treated with various dosages of DOP (0, 5, 10, 25, 50, 100 and 250 μg/ml) for 48 hours. Cell viability analysis showed that DOP at concentrations from 0 to 100 μg/ml had no significant effect on the cell viability (Figure 2A). DOP only at high concentration, 250 μg/ml, suppressed the cell viability (*p* < 0.05). For the safety, a DOP concentration of 50μg/ml was chosen the formal study. For evaluation of the effect of DOP in HG-induced MPC5 cells, cells were cultured with 30mM of D-glucose and DOP for 48 h, and then were stimulated with insulin. Afterwards, glucose consumption, productions of MDA, as well as the phosphorylation of IRS-1 and AKT were respectively determined. Results shown in Figure 2A and 2B demonstrated that DOP treatment distinctly relived HG-induced reduction of glucose consumption (*p* < 0.001, Figure 2B) and increase of MDA production (*p* < 0.001, Figure 2C), while no notable difference was observed at the initial time. Besides, DOP treatment notably reversed HG-triggered suppressing effects on the expression of IRS-1 (phospho Y632), AKT (phospho Ser473), AKT (phospho Thr308) and Nephrin (*p* <0.05 or *p* <0.001, Figure 2D, 2E). Moreover, detection of γ-H2A.X foci formation, an indicator of DNA double strand breaks (DSBs), revealed that HG treatment distinctly augmented the percentage of cells bearing γ-H2A.X foci (*p* <0.01, Figure 2F), while this effect was notably reversed by DOP treatment (*p* <0.01, Figure 2F). These outcomes manifested that DOP treatment distinctly relieved HG-induced oxidative stress and IR, as well as DNA damage in MPC5 cells.

### DOP treatment ameliorated HG-induced oxidative stress, IR and DNA damage of MPC5 cells by phosphorylation of IRS1/AKT axis

**Figure 3:**
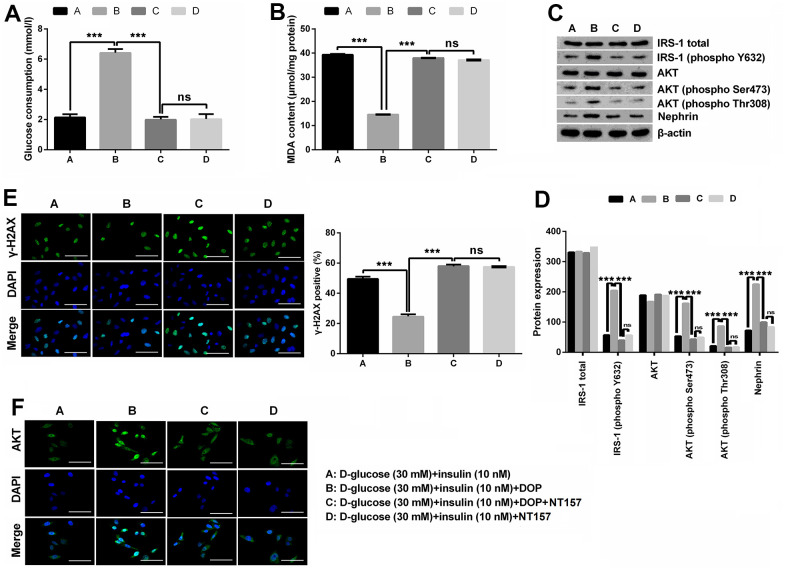
**DOP treatment ameliorated HG-induced oxidative stress, IR and DNA damage of MPC5 cells by activating the phosphorylation of IRS-1/AKT.** (**A**, **B**) IRS-1/2 inhibitor NT157 treatment remarkably impeded DOP-induced protective effects on HG-treated MPC5 cells, exhibiting as markedly decreased glucose consumption and increased malondialdehyde (MDA) generations. (**C**, **D**) IRS-1/2 inhibitor NT157 treatment notably reversed HG-induced increased phosphorylation of IRS-1 (phospho Y632), AKT (phospho Ser473), AKT (phospho Thr308) and Nephrin. (**E**) IRS-1/2 inhibitor NT157 treatment distinctly augmented DNA damage, exhibiting as markedly increased the formation of γ-H2A.X foci compared with DOP treated group. The bar in the figure indicates 10 μm. (**F**) DOP treatment remarkably increased the nuclear translocation of AKT, while this effect was notably counteracted by IRS-1/2 inhibitor NT157 treatment. The bar in the figure indicates 10 μm (*p < 0.05; ** *p* < 0.01; *** *p* < 0.001, n=3).

NT157 is a selective inhibitor of IRS-1/2. NT157 exposure was reported to elicit time- and dose-dependent reduction in IRS-1 levels, and suppress the activation of IGF1R, as well as IGF1-induced activation of AKT. In this study, for exploration of the potential mechanisms of how DOP treatment relieved HG-induced effects, MPC5 cells were precultured with IRS-1/2 inhibitor NT157. NT157 treatment remarkably reversed DOP treatment-induced protective effects on MPC5 cells, exhibiting as markedly decreased glucose consumption (*p* < 0.001, Figure 3A), while notably augmented MDA productions in MPC5 cells (*p* < 0.001, Figure 3B). Besides, NT157 treatment also remarkably reversed DOP-induced increased phosphorylation of IRS-1 (phospho Y632), AKT (phospho Ser473), AKT (phospho Thr308) and Nephrin (all *p* < 0.01, Figure 3C, 3D). As for the formation of γ-H2A.X foci, the results displayed in Figure 3E demonstrated that NT157 treatment distinctly augmented the number of γ-H2A.X positive cells compared with DOP treated group (*p* < 0.001), which indicates that DNA damage still exists after treated with IRS-1/2 inhibitor NT157. Previous investigations revealed that the unphosphorylated AKT is mainly located in the cytoplasm, while AKT phosphorylation induces the translocation to the nuclear, thereby activating the downstream pathways. Moreover, DOP treatment remarkably increased the nuclear translocation of AKT, while this effect was notably counteracted by IRS-1/2 inhibitor NT157 treatment (Figure 3F), which indicating that DOP treatment promoted the activation of AKT, while IRS-1/2 inhibitor NT157 repressed the activation of AKT. Besides, there was no significant difference observed between the DOP plus NT157 and NT157 group (Figure 3A–3F). Combination of these outcomes demonstrated that DOP treatment relieved HG-triggered injury through enhancing the phosphorylation of IRS1/AKT.

### HG stimulation impaired mitochondrial function in MPC5 cells

**Figure 4:**
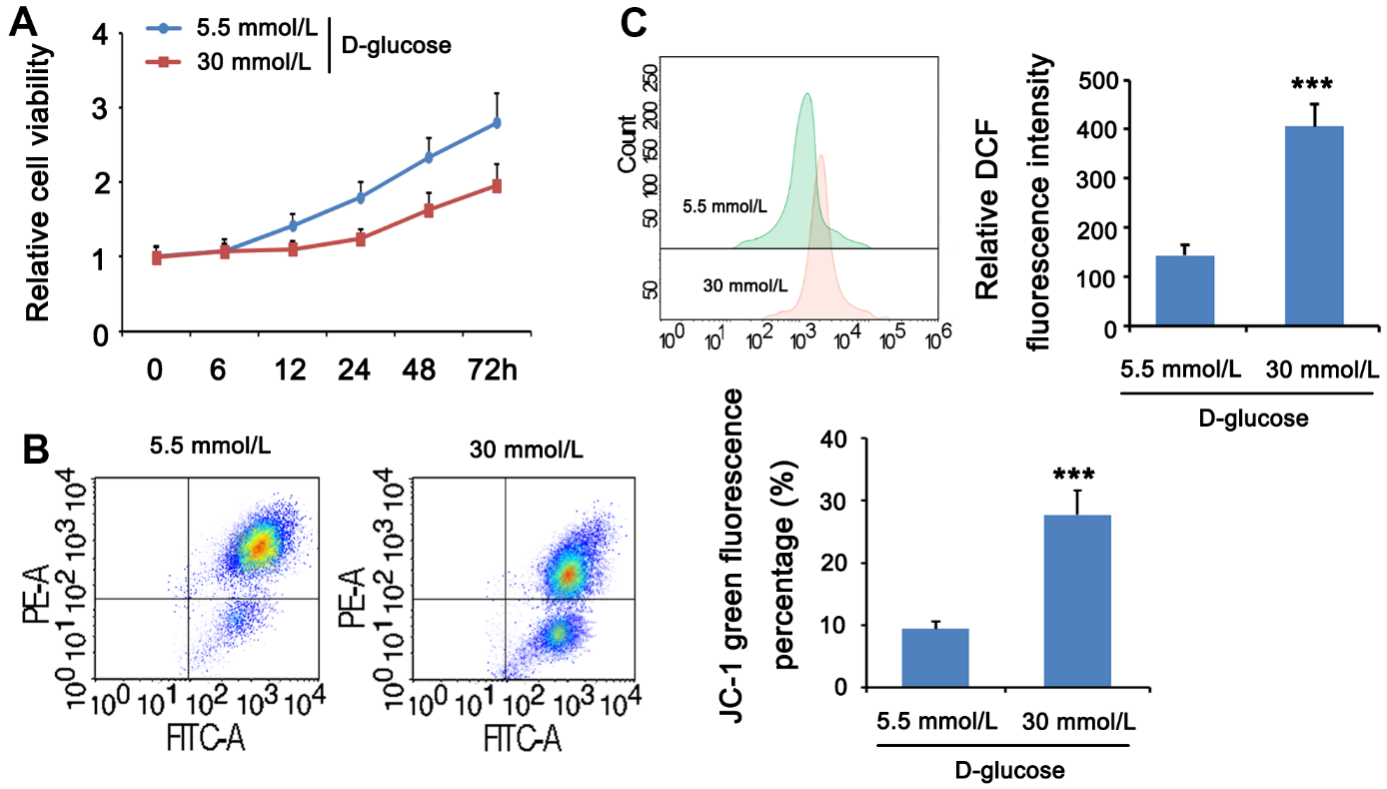
**HG stimulation impaired mitochondrial function in MPC5 cells.** MPC5 cells were divided into 5.5mm D-glucose group and 30mM D-glucose group. (**A**) Viability of MPC5 cells was detected by CCK-8 assay. From 12 h after stimulation, cell viability in the 30mM D-glucose group was significantly lower than that in the 5.5mm D-glucose group. (**B**) Mitochondrial membrane potential was evaluated by JC-1 staining assay. The percentage of mitochondrial membrane potential (Δψm) decreased is the percentage of JC-1 monomer. (**C**) DCF fluorescence was used to detect ROS levels. (*** *p* < 0.001; n=3).

HG can cause cell and mitochondrial damage. To investigate the mitochondrial activity of MPC5 cells stimulated with HG, we measured cell viability, MMP, and ROS levels in both normal and HG groups. The CCK-8 assay revealed that the normal glucose group’s cell viability improved with time, whereas the high glucose group’s cell viability decreased and was lower than the comparable time point of the normal glucose group from 12 h. (Figure 4A). JC-1 labeling was used to assess the effect of HG on mitochondria in MPC5 cells by measuring MMP. The intensity ratio of JC-1 aggregates to JC-1 monomers or the percentage of JC-1 green fluorescence, which shows the change of MMP, was considerably lower in the HG group than in the normal glucose group (*p* <001, Figure 4B). ROS production was then evaluated by flow cytometry with DCFH-DA labeling to determine the ROS levels in MPC5 cells following stimulation with HG. Figure 4C. reveals that the relative DCF fluorescence intensity was considerably greater in the HG group than in the control group, showing that HG stimulation generated an excess of ROS generation (*p* <0.001, Figure 4C). These findings imply that a HG concentration may result in mitochondrial malfunction, increased ROS, and lower cell viability.

### DOP enhanced mitophagy in MPC5 cells

**Figure 5:**
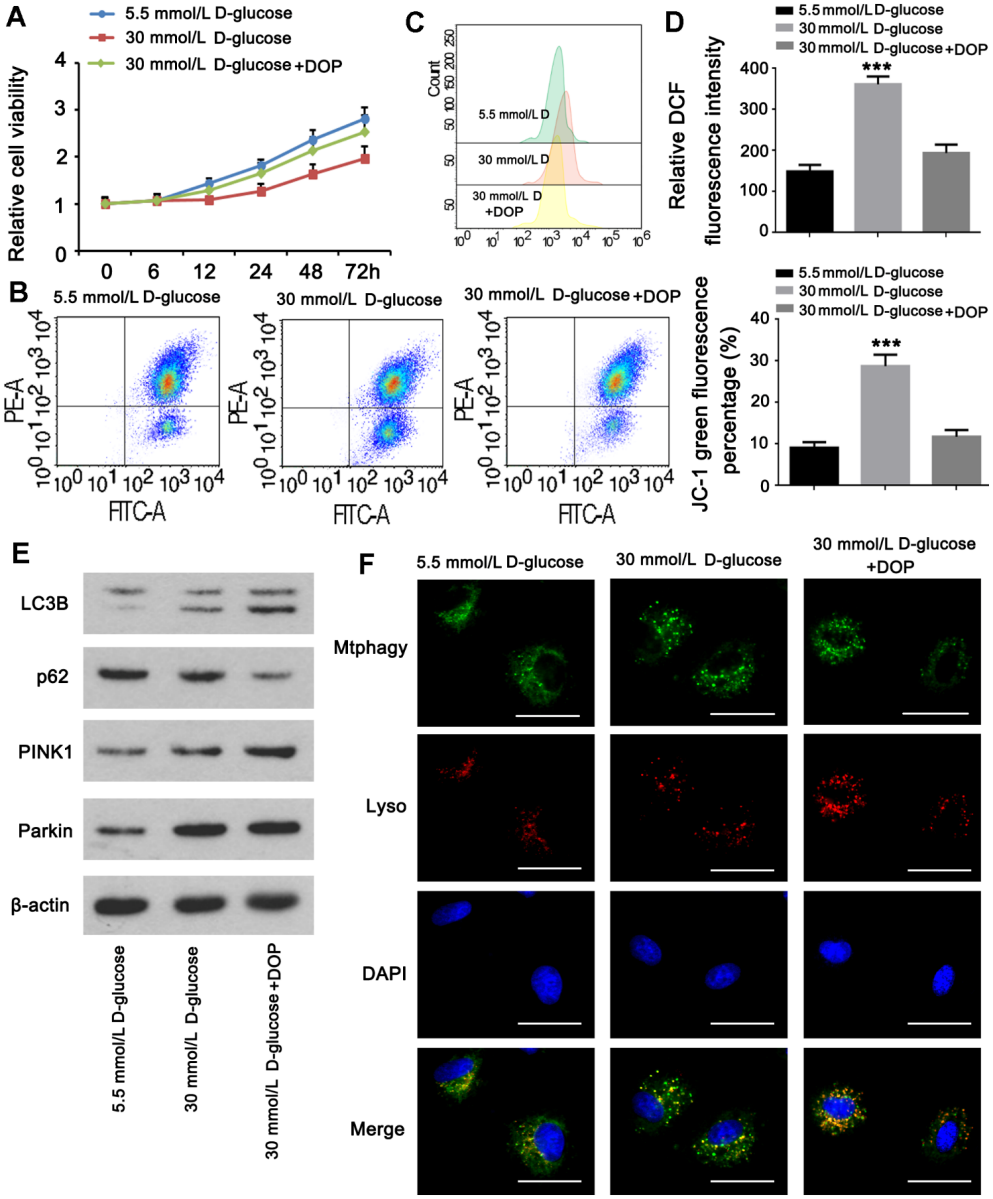
**DOP elevated mitophagy in MPC5 cells.** MPC5 cells were divided into 3 groups: normal glucose (5.5mM), HG (30mM) and HG with DOP treatment. (**A**) MPC5 cell viability of the three groups was detected by CCK-8 assay. (**B**) Flow cytometry was conducted to observe the mitochondrial membrane potential changes using JC-1 staining. (**C**, **D**) Detection of ROS content by using flow cytometry. (**E**) The expression of mitophagy-related proteins (Parkin, PINK1, LC3B) and mitophagy receptor P62 were detected by western blot. (**F**) Fluorescence intensity of mitochondrial MtphagyTracker and LysoTracker. The bar in the figure indicates 8 μm. (** *p* < 0.01; *** *p* < 0.001; n=3).

Experiments were done to determine whether DOP may stimulate mitophagy in MPC5 cells *in vitro.* The CCK-8 assay revealed that the viability of cells in group A was normal, whereas the viability of cells in group B was diminished. Compared to group B, the cell viability of group C was greatly restored. HG generated a pronounced decline in cell viability, which was reversed by DOP administration (Figure 5A). Treatment with DOP can attenuate mitochondrial dysfunction generated by HG levels (*p* <0.001, Figure 5B). Flow cytometry revealed that the ROS level in the HG group was substantially higher than in the control group. In contrast, ROS levels were considerably lower in the DOP-treated group versus the HG group (*p*<0.001, Figure 5C). Mitophagy has a protective effect on cells. Using western blotting, the expression of mitochondrial mitophagy-related proteins was identified. After DOP stimulation, PINK1, Parkin, and LC3B were up-regulated, while the autophagy receptor P62 was down-regulated (Figure 5E). HG stimulation and DOP increased the fluorescence intensity of mitophagy and lysosomes in comparison to normal glucose (Figure 5F).

### DOP alleviated the injury of podocytes in the diabetic mice

**Figure 6:**
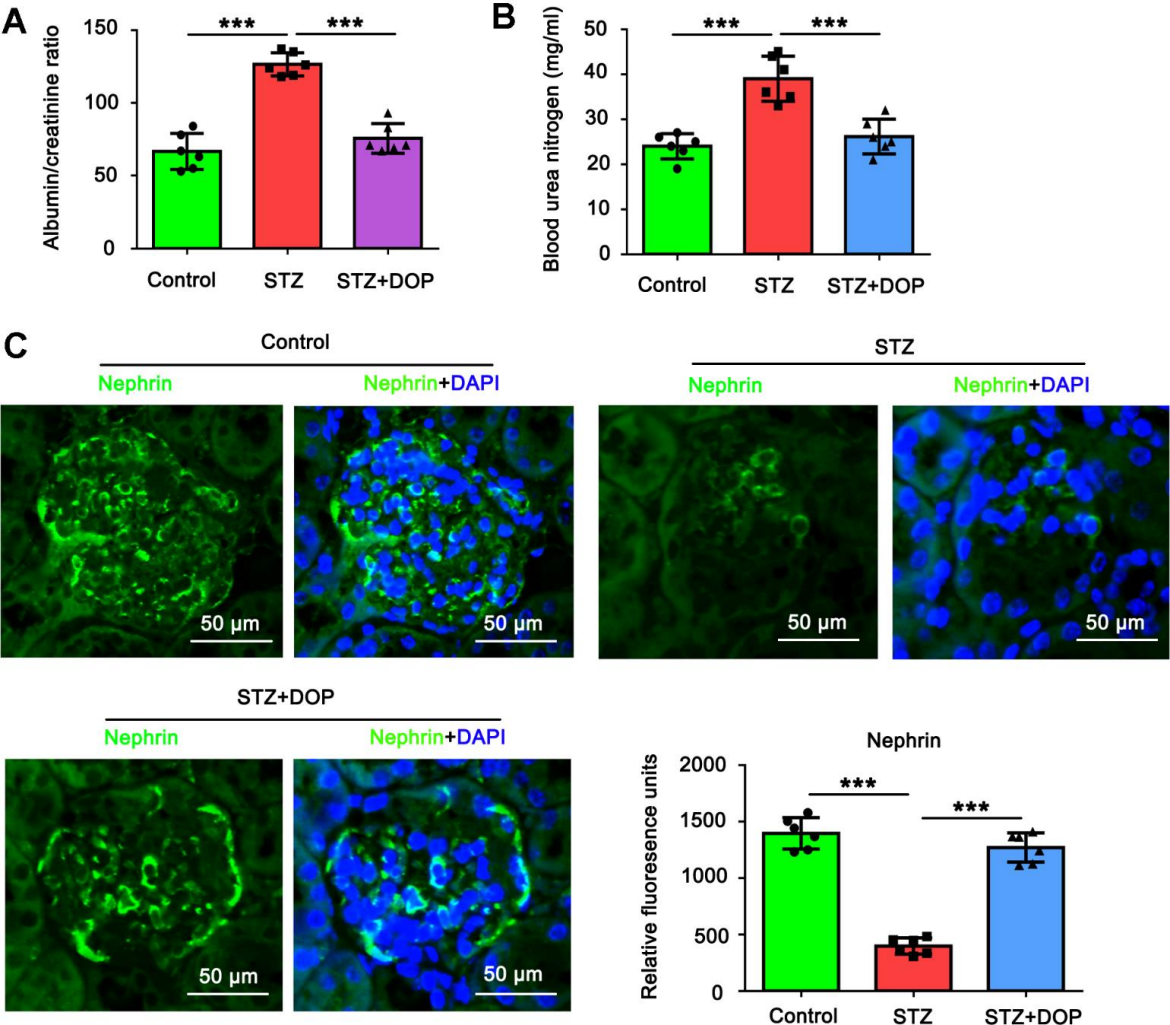
**DOP alleviated the injury of podocytes in the diabetic mice.** In this study, C57BL/6 mice were randomly allocated to three groups: control, STZ and STZ+DOP groups with six mice in each group. In STZ and STZ+DOP groups, C57BL/6J mice were injected intraperitoneally with STZ for 5 consecutive days. Two weeks after diabetes was induced, the mice in STZ+DOP group were injected with DOP (10mg/kg) intraperitoneally once a day for eight consecutive weeks. After the ten weeks, the blood was retained and the urine during 24h was taken from all the mice. (**A**) The urine albumin and blood creatinine were assessed for the evaluation of the albumin/creatinine ratio. (**B**) Blood urea nitrogen (BUN) was determined using the Urea Nitrogen (BUN) Colorimetric Detection Kit. (**C**) Immunofluorescence analysis of renal tissue was conducted to assess Nephrin protein level (*** p < 0.001; n=6).

In this study, we established diabetes model in mice by repeated injection with STZ. Compared to the control mice, both the albumin/creatinine ratio and blood urea nitrogen were increased in the diabetic mice (both *p*<0.001, Figure 6A, 6B). In addition, as indicated by the immunofluorescence analysis of renal tissue, the level of Nephrin in podocytes was decreased in the diabetic mice (both *p*<0.001, Figure 6C). All these data together suggested the injury of podocytes in the diabetic mice. However, intraperitoneal injection with DOP reversed the increase of the albumin/creatinine ratio and blood urea nitrogen in the diabetic mice. Furthermore, the level of Nephrin in podocytes was restored in the diabetic mice. Therefore, DOP alleviated the injury of podocytes in the diabetic mice.

## DISCUSSION

In this research, we primarily investigated the protective efficacy of DOP and its potential regulating mechanism in DN. The results demonstrated that DOP treatment effectively relieved HG-induced oxidative stress, IR and DNA damage, and promoted mitophagy of podocyte *in vitro*. Our research might provide theoretical basis of DOP for clinical treatment of DN.

In recent years, emerging investigations have discovered that HG-induced inflammation and oxidative stress play important roles in the progression of IR, which subsequently impacted protein metabolism [28–30]. Besides, oxidative stress could bring about cellular disruption and damage in eukaryotic cells [31], exhibiting as elevated ROS-induced cellular macromolecules damage through modification of the targets, such as DNA, proteins, lipids and so on [32]. Moreover, Tatsch et al. reported that oxidative DNA damage was tightly associated with IR in diabetes [33]. Considering that numerous researchers have investigated DN by treating podocytes or rats with hyperglycemia [34, 35], we performed HG induction on MPC5 cells in this study. Our results displayed that the glucose consumption was distinctly reduced, while ROS and MDA generations were markedly augmented by HG inducement, which was consistent with previous published literatures [36, 37]. Besides, the phosphorylation of IRS-1, AKT and expression of Nephrin were all notably increased by HG inducement, which could be explained by the results of earlier studies [38]. These outcomes manifested that the glomerular podocyte IR model was successfully established. These results were in line with our findings, which demonstrated that DOP treatment notably relieved HG-induced repressing effects on glucose consumption and phosphorylation of IRS-1/AKT, as well as the promoting effects on the generations of ROS, MDA and γ-H2A.X. Collectively, these outcomes manifested that DOP treatment remarkably alleviated HG-induced oxidative stress and IR.

Mitophagy is a protective process that maintains cellular homeostasis by removing damaged cellular debris, for example, mitophagy protects beta cells from inflammatory injury in diabetic patients [39] and protects nerve cells from cerebral ischemia-reperfusion injury [40]. Mitophagy can reduce neuronal apoptosis through MT2/Akt/NF-κB pathway under HG environment [41]. The role of mitophagy in podocyte injury in diabetic nephropathy has been gradually recognized [23, 42]. HG-induced advanced glycation end products induce ROS production and mitophagy [22, 43]. Furthermore, mitophagy can be activated by oxidative stress, and mitophagy can reduce ROS levels [21]. Therefore, increasing mitophagy levels in podocytes may reduce ROS and thereby reduce IR.

However, HG can induce mitophagy in podocytes and play a certain degree of protection, but HG can also inhibit mitophagy through AMPK/mTOR pathway. We observed that podocyte viability was significantly inhibited under HG conditions, accompanied by an increase in the level of mitophagy, but HG-induced mitophagy was insufficient to counter HG-induced cell damage [44, 45]. Therefore, it is necessary to find ways to improve mitophagy. Icariin [46], Astragaloside II [47], and Orientin [21] were previously found to regulate podocyte mitophagy, but their roles in IR are not clear. DOP has various effects of anti-oxidative stress [48], anti-inflammatory response [49], inhibiting apoptosis [50], and anti-tumor. Here, for the first time, we found that DOP can effectively reduce oxidative stress in podocytes under HG conditions, thereby effectively reducing IR, possibly by significantly enhancing PINK1/parkin pathway-mediated mitophagy. Intriguingly, in colon cancer CT26 cells, DOP stimulated ROS production and reduced mitochondrial membrane potential (MMP) by activating AMPK/mTOR autophagy signaling, and finally disrupted mitochondrial function [51]. Recent studies have also found that mitophagy-mediated adipose inflammation can promote IR in the liver [52, 53]. The above results indicate that there are heterogeneity and specificity in the role of DOP in different cell types. This effect of DOP in podocytes may directly enhance mitophagy, and the specific mechanism involved needs to be further elucidated in subsequent studies. In addition, our research results have clinical promotion value, suggesting that DOP may not be used solely in IR, which requires more exploration.

In conclusion, this research discovered that DOP treatment relieved HG-triggered oxidative stress and IR, and promoted mitophagy processes through regulation of PINK1/parkin pathway.

## MATERIALS AND METHODS

### Extraction of DOP

DO was used for the extraction of crude DOP employing the water extraction and alcohol precipitation method. The refined crude DOP was obtained after series of procedures including decolorizing, repeated freezing and thawing, dialysis, G50 gel chromatography, and freeze-drying. Following experiments validated that DOP has a sugar content of 105%, a molecular weight is 1.264×10^5^ Da, and a uniform molecular weight, and almost free of nucleic acid and proteins. Besides, it is composed of glucomannan. These results demonstrated that the properties of the obtained DOP are well and meets the requirement for following experiments.

### Cell culture and treatment

The mouse podocyte clone 5 (MPC5) cells were obtained from the Gaining Biological company (No. CM-M121, Shanghai, China). Cells were cultured in Dulbecco’s modified Eagle’s medium (DMEM, Solarbio, Beijing, China) containing 10% fetal bovine serum (FBS, Gibco, Australia) and 1% penicillin-streptomycin solution (Beyotime, Shanghai, China) under 5% CO_2_ and 37° C conditions. The culture medium was refreshed every 3 days. To determine the time course of HG on glucose consumption, ROS and MDA production, insulin receptor substrate (IRS)-1 and protein kinase B (AKT) phosphorylation, as well as Nephrin expression, MPC5 cells were cultured with normal or high D-glucose (5.5mM or 30mM) [22] for 1 h, 3h, 6 h, 12 h, 24 h, 48 h and 72 h, respectively. After that, cells were stimulated with insulin (10 nM, Aladdin, Shanghai, China) for 15 min, and then following assays were conducted. For evaluation of the protective effect of DOP against HG-induced injuries, MPC5 cells were cultured with normal/high D-glucose (5.5mM/30mM) and DOP (50μg/ml, the concentration was based on the results from cell viability assay) for 48 h, and then were stimulated with insulin (10 nM) for 15 min for following experiments. Additionally, for exploration of the potential regulating mechanism of DOP, MPC5 cells were preincubated with IRS-1/2 inhibitor NT157 (2 μM; MedKoo Biosciences, NC, USA) for 2 h before DOP treatment.

### Glucose uptake assay

After treatment, the glucose uptake in MPC5 cells was determined by glucose oxidase-peroxidase (GOD-POD) method. MPC5 cells were seeded on 6 well plates and maintained in a humidity 37° C incubator for 48 h. Then, the supernatant was collected and ice-cold KRP buffer washing was performed to terminate glucose uptake. Thereafter, cell lysate was obtained after freezing and thawing the cells, and glucose estimation was subsequently conducted. Then, calculation of glucose uptake was performed through determining the difference between the final and initial glucose content in the collected medium using GOP-POD method. Briefly, 10 μl of collected samples and 1 ml of prepared reagent was mixed and incubated at 37° C for 15 min. Finally, the absorbance of the sample (A_sample_) and standard (A_standard_) was respectively determined with the help of a spectrophotometer (HACH, Shanghai, China). The glucose concentration was calculated following the formula listed below: Glucose concentration mmol/l = A_sample_/A_standard_ × Concentration_standard_.

### ROS determination assay

The ROS level in MPC5 cells was determined by employing ROS assay kit (Beyotime). After treatment, the culture medium was replaced with diluted 2,7-dichlorodihydrofluorescein diacetate (DCFH-DA) reagent, and then the cells were incubated at 37° C for 20 min. After rinsing with serum-free DMEM medium, cells were collected and the fluorescence intensity was determined employing a fluorescence microplate reader (BioTek Instruments, VT, USA) at excitation/emission wavelengths of 488 and 525 nm.

### Malondialdehyde (MDA) determination assay

The MDA content was determined by the Lipid Peroxidation MDA Assay Kit (Beyotime) following the manufacturer instructions. After treatment, MPC5 cells were rinsed, lysed with lysis buffer and centrifuged. Then, the supernatant was collected and used for subsequent MDA detection. In brief, the standards were firstly diluted to a final concentration of 1, 2, 5, 10, 20 and 50 μM for preparation of the standard curve. Afterwards, 0.1 ml of the prepared supernatant and 0.2 ml of MDA detection solution was mixed and heated at 100° C for 15 min. After cooling to room temperature and centrifuged at 1000 × g for 10 min, the supernatant was collected. A microplate reader (Bio-Rad, CA, USA) was employed for determination of MDA absorbance at 532 nm. Additionally, the protein concentration in the supernatant was determined employing the BCA protein assay kit (Beyotime).

### Western blot

After treatment, extractions of total cellular proteins were carried out utilizing the RIPA lysis buffer (Beyotime) with the presence of protease inhibitor cocktail (Beyotime). Determination of protein concentration was fulfilled employing the BCA protein assay kit. Then, analysis of interested proteins was performed using SDS-PAGE. After electrophoresis, proteins were transferred onto the nitrocellulose (NC, Pierce, IL, USA) membranes and blocked in 5% skim milk powder at 37° C for 2 h. After rinsing with TBST, the membranes were incubated with primary antibodies, including anti-IRS-1 total (1:1000, #ab40777, Abcam, MA, USA), anti-IRS-1 (phospho Y632) (1:1000, #ab109543, Abcam), anti-IRS-1 (phospho S312) (1:500, #ab4865, Abcam), anti-IRS-1 (phospho Y896) (1:500, #ab4873, Abcam), anti-IRS-1 (phospho S527) (1:200, #ab65745, Abcam), anti-IRS-1 (phospho S616) (1:1000, #ab4776, Abcam), anti-Nephrin (1:500, #GTX31936, GENETEX, TX, USA), anti-AKT (1:400, #OM238722, Omnimabs, CA, USA), anti-AKT (phospho Ser473) (1:500, #OM270698, Omnimabs), anti-AKT (phospho Thr308) (1:500, #OM238718, Omnimabs), anti-PINK1(1:500, #ab186303, Abcam), anti-Parkin(1:500, #ab77924, Abcam), anti-LC3B(1:500, #ab192890, Abcam) and anti-β-actin (1:2000, #66009-1-Ig, Ptgcn, IL, USA) at 4° C overnight. Afterwards, the membranes were rinsed with TBST and maintained with HRP-conjugated secondary antibodies (1:4000) at room temperature for 1 h. Target protein signals were developed with the enhanced chemiluminescence reagent (ECL, Pierce) and analyzed with the ImageJ software (National Institutes of Health, MD, USA).

### Immunofluorescence (IF) analysis

The γ-H2A.X foci formation and location of AKT was determined employing IF. MPC5 cells were grown on cover-slips. After treatment, cells were respectively fixed in 4% paraformaldehyde (PFA), permeabilized with 0.2% Triton X-100 and blocked with goat serum for 10 min, 5 min and 1 h. Then, primary antibodies, including anti-gamma-H2A.X antibody (Cell Signaling, MA, USA) and anti-AKT antibody (#OM238722, Omnimabs, CA, USA), incubations were carried out at 4° C overnight. After rinsing with PBS, Alexa Fluor® 488-conjugated secondary antibody incubation was performed at room temperature for 1 h. Nuclei counterstaining was performed with DAPI. Images were obtained with an Olympus microscope (DP72) and analyzed utilizing the Image-pro plus 6.0 software. Numbers of normal cells and damaged cells were respectively counted and the cell damage ratio was calculated.

### CCK-8 assay

The Cell Counting Kit-8 (CCK-8, Dojindo Laboratories, Kumamoto, Japan) was used to analyze cell viability in accordance with the manufacturer’s instructions. MPC5 cells were seeded and grown at a density of 5×10^3^/well in 100 μL of DMEM (Solarbio, China) in 96-well microplates (Corning, USA). The MPC5 cells were then grown with low or high concentrations of D-glucose (5.5mM/30mM). In addition, MPC5 cells were treated with various dosages of DOP (0, 5, 10, 25, 50, 100 and 250 μg/ml) for 48 hours. CCK-8 reagent (10μL) was added to each well, followed by two hours of culture. Each experiment was conducted in triplicate. The absorbance was measured at predetermined time intervals (0, 6, 12, 24, 48, 72 h) and analyzed at 450 nm using a microplate reader (Bio-Rad, CA, USA) with wells containing no cells serving as blanks. Absorbance served as an indicator of cellular growth.

### Detection of mitochondria membrane potential (MMP)

Using a mitochondrial membrane potential assay kit with JC-1 (Beyotime, Beijing, China) according to the manufacturer’s instructions, the mitochondrial stability was evaluated. MPC5 cells were grown in 6-well plates for 24 hours before being treated with normal or high D-glucose (5.5mM/30mM) and DOP. The cells were treated with JC-1 fluorescent dye in the dark at 37° C for 20 minutes. The cells were then rinsed twice with JC-1 staining buffer, followed by a 10-minute Hoechst treatment. In mitochondria with normal membrane potentials, JC-1 aggregates glow red, whereas in mitochondria that have been injured and depolarized, JC-1 monomers fluoresce green. The ratio of the intensity of JC-1 aggregates to the intensity of JC-1 monomers was utilized to monitor the change in mitochondrial membrane potential. Flow cytometry was then utilized to determine relative fluorescence.

### Mitophagy assay

The Mitophagy detection kit (Cat.#MD01, Dojindo Molecular Technologies, Kumamoto, Japan) includes MtphagTracyker and LysoTracker for monitoring mitophagy. According to the manufacturer’s directions, cells were grown in confocal dishes and treated with MtphagTracyker and LysoTracker for 30 minutes. MtphagyTracyker (green) and LysoTracyker (red) were co-localized with mitophagy-indicating yellow dots. Fluorescence was measured three times for each group using a confocal fluorescence microscope (IX81-FV1000, Olympus, Markham, ON, Canada). Using NIH Image J, the fluorescence intensity of co-localized yellow dots was measured.

### Animal study

C57BL/6 mice (age:5–6 weeks old; weight: 18–22 g) were obtained from Zhejiang Vital River Laboratory Animal Technology Co., Ltd (Zhejiang, China). Animals were randomly allocated to three groups: control, STZ and STZ+DOP groups with six mice in each group. The study was performed using blinded method during analysis of every indicator of animals. STZ (Sigma-Aldrich, Shanghai, China) was dissolved in sodium citrate buffer (pH=4.5) to a concentration of 8 mg/mL. C57BL/6J mice were deprived of food overnight and then injected intraperitoneally with 40 mg/kg STZ for 5 consecutive days. The control mice were injected with an equivalent volume of sodium citrate buffer. The fasting blood glucose level in the tail vein of all experimental mice was measured with a blood glucose meter (OneTouch Verio Vue, OneTouch, Shanghai, China). The diabetes model was successfully developed when the blood glucose was more than ≥ 13.3 mmol. Two weeks after diabetes was induced, the mice were injected with DOP (10mg/kg) intraperitoneally once a day for eight consecutive weeks. After the ten weeks, the blood was retained and the urine during 24h was taken. Urine albumin was assessed using a mouse albumin ELISA kit (Bethyl Lab, Hamburg, Germany). Creatinine was measured using the Creatinine Assay Kit (ab204537, Abcam, MA, USA). Blood urea nitrogen (BUN) was determined using the Urea Nitrogen (BUN) Colorimetric Detection Kit (Thermo Fisher Scientific, MA, USA). Under the pentobarbital sodium anesthesia of the mice, the kidneys were obtained via the surgery for subsequent experiments.

### Statistical analysis

All data were shown as mean ± SEM. Each experiment has at least three replications. GraphPad Prism software was used for statistical analysis. One-way analysis of variance (ANOVA) or Student’s *t*-test was used for data analysis. *P* < 0.05 represents statistically significant.

### Availability of data and material

The datasets generated/analyzed in the present study are available upon reasonable request from the corresponding author.

### Consent for publication

The consent for publication was obtained from all participants of this study.
